# Economy in the Patients' Diet

**Published:** 1912-06-22

**Authors:** 


### INSTITUTIONAL HOUSEKEEPING.

### Economy in the Patients' Diet.

Under the multiple system of control which prevails in far too many hospitals responsibility for economy in the food of the patients is divided among three different sets of persons. The steward or secretary, together with the weekly board, are responsible for making contracts and controlling supplies; the resident medical staff order the diets; the matron or housekeeper makes the best of the supplies under her control and supervises the preparation of whatever may be ordered. Although the ingredients of which the patients' diet consists are very simple, yet under these conditions there is considerable risk of loss and extravagance at each stage.

The articles of diet most in use for patients are eight in number: milk, meat, fish, bread, butter, rice, eggs, and tea. We have recently been favoured with particulars of the actual cost of the patients' diet in a London general hospital. The cost for one week of 56 male patients in one surgical ward was £1 12s. 0½d., or an average of 4s. 1d. a head; in another surgical ward for 53 male patients the cost was £1 6s. 11½d., or an average of 3s. 7¾d. a head. In a medical ward the total for 55 patients was £1 14s. 5½d., the average being 4s. 6½d., and the medical wards, we were told, averaged on the whole considerably higher than the surgical. These amounts were for the articles we have named, the cost of other articles amounting to barely a shilling a week for the entire ward. The field is therefore limited; but, on the other hand, where the quantities used are so large the smallest deviation from economy in procuring supplies tells impressively in the totals. In this particular hospital the price of milk alone has risen 15 per cent., and the result has been instantly felt in the averages. But it is a notable fact that the difference in the cost of common necessaries from one year to another is negligible as compared with the difference in price paid for these articles between one hospital and another. Not till supplies have been secured at the lowest market price consistent with good quality can any hospital be said to have laid the basis of economy in this branch of the commissariat.

The next stage lies in ordering the diets, and unhappily this lies at the discretion of young resident officers who, whatever their skill in medicine, often have all to learn in the gentle art of economy. That they get very little assistance in learning this art in some establishments is too well known to need comment. But in others a laudable attempt is made to impart to them the great secret of hospital administration, which consists in getting the best value for the money in every single department of the work. We have been much edified by reading the little pamphlet circulated by the London Hospital among "residents, clinical assistants, clerks, dressers, and nurses" with a view to dissipating the common ignorance about the value of things in common use. There is a list of the cost of "extras" per diet, with practical applications, from which we quote the following:—

Suppose that a patient on bread and milk is given two eggs; the price rises from 4.5d. to 6.3d. If to the same diet one pint of beef-tea is added, "because the patient does not like his milk," the price rises from 4½d. to 8½d. Let us take another instance. It is a common practice to order bread and milk diet with two eggs and chicken. This costs nearly 10d., and the patient receives—3 pints of milk, 6 oz. bread, pudding, chicken, 2 eggs. This is expensive and is not a satisfactory diet from the patient's point of view. Much waste is caused by leaving a patient on some expensive built-up diet long after he could have been better fed on one of the hospital diets . . . . Do not order cream if the patient can take other forms of fat, such as ol. nutriens. Even ol. nutriens c. malt in therapeutic doses is cheaper than cream. Be sparing with bacon which adds 3d. to a diet and can be used for breakfast only. Frequently a patient is ordered—bread and milk, 2 eggs, bacon, mince. This costs nearly 1s., and is less varied and efficient than No. 4 diet, which costs under 8d. To show what can be done by careful management, a slight drop in the following diets—No. 4, chicken and bacon—which occurred in 1907 produced a saving of £334.

This is the way to bring responsibility home.

When all is done it is on the housekeeper that the weight of the burden must fall, for it is on her that it depends to provide that no waste occurs in the kitchen, that the patient gets the full amount which has been ordered for him, that he gets it so cooked as to be readily digested by his somewhat enfeebled system, and served in appetising fashion. Supposing that his 6 oz. of beef reach him greasy and leathery, or that half of it is sinew and fat, or that his fish is swimming in cold water, or that his egg is stale, or that his rations of bread have been lying on a plate for hours till they are dry and brittle, or that the longed-for cup of tea is cold and bitter, or that the rice lies hard at the bottom of the pudding: such "accidents" mean that the diet is surely wasted, although it makes no figure in the hospital accounts. Good organisation is taxed to the utmost to get the food to the patients in first-rate condition, and none understand the difficulties but those who have tried to master them. Yet if many of the housekeeper's triumphs are buried in the depths of her own domain this is one which will bring her at once into fame. The secret of making the hospital popular and spreading a sense of satisfaction through the entire establishment lies in giving the patients appetising meals. Having regard to the purpose for which the patient has entered the wards, it is the housekeeper's *rôle* which must after all be reckoned of the greatest importance in the difficult art of economy in diet.

---

### To Our Readers.

These columns are open to queries and discussion touching all domestic matters. Matrons are invited to send us the result of any experiments they are making with a view to promoting the comfort of the staff. Those who are dissatisfied in regard to the quality of the goods supplied on contract can have samples tested by forwarding them to the Editor, marked "Institutional Housekeeping." Estimates given of the cost of boarding patients, nurses, and staff.

